# Prevalence, determinants and systems-thinking approaches to optimal hypertension control in West Africa

**DOI:** 10.1186/1744-8603-10-42

**Published:** 2014-05-21

**Authors:** Juliet Iwelunmor, Collins O Airhihenbuwa, Richard Cooper, Bamidele Tayo, Jacob Plange-Rhule, Richard Adanu, Gbenga Ogedegbe

**Affiliations:** 1 Department of Kinesiology and Community Health, University of Illinois, Urbana-Champaign, 123 Huff Hall, 1206 S. Fourth St, Champaign, IL 61820, USA; 2 Department of Biobehavioral Health, The Pennsylvania State University, 219 Biobehavioral Health Building, University Park, PA 16802, USA; 3 Department of Public Health Sciences, Loyola University Medical Center, 2160 S. First Ave, Maywood, IL 60153, USA; 4 School of Medical Sciences, College of Health Sciences, Kwame Nkrumah University of Science and Technology, Kumasi, Ghana; 5 School of Public Health, College of Health Sciences, University of Ghana, P.O. Box LG13, Accra, Ghana; 6 Center for Healthful Behavior Change, Division of General Internal Medicine, Department of Medicine, New York University School of Medicine, New York, USA

## Abstract

**Background:**

In West Africa, hypertension, once rare, has now emerged as a critical health concern and the trajectory is upward and factors are complex. The true magnitude of hypertension in some West African countries, including in-depth knowledge of underlying risk factors is not completely understood. There is also a paucity of research on adequate systems-level approaches designed to mitigate the growing burden of hypertension in the region.

**Aims:**

In this review, we thematically synthesize available literature pertaining to the prevalence of hypertension in West Africa and discuss factors that influence its diagnosis, treatment and control. We aimed to address the social and structural determinants influencing hypertension in the sub-region including the effects of urbanization, health infrastructure and healthcare workforce.

**Findings:**

The prevalence of hypertension in West Africa has increased over the past decade and is rising rapidly with an urban-rural gradient that places higher hypertension prevalence on urban settings compared to rural settings. Overall levels of awareness of one’s hypertension status remain consistently low in West African. Structural and economic determinants related to conditions of poverty such as insufficient finances have a direct impact on adherence to prescribed antihypertensive medications. Urbanization contributes to the increasing incidence of hypertension in the sub-region and available evidence indicates that inadequate health infrastructure may act as a barrier to optimal hypertension control in West Africa.

**Conclusion:**

Given that optimal hypertension control in West Africa depends on multiple factors that go beyond simply modifying the behaviors of the individuals alone, we conclude by discussing the potential role systems-thinking approaches can play to achieve optimal control in the sub-region. In the context of recent advances in hypertension management including new therapeutic options and innovative solutions to expand health workforce so as to meet the high demand for healthcare, the success of these strategies will rely on a new understanding of the complexity of human behaviors and interactions most aptly framed from a systems-thinking perspective.

## Background

West Africa comprises 16 countries that are highly diverse both in ethnicity and culture. For example, the region is characterized by numerous cultural and ethnic affiliations that govern the language people speak, how they interact or even what food they eat [1]. However, the region also shares general similarities for example with food preparation with salt and bouillon cubes such as Maggi cubes playing a central role in food preparation and preservation in West Africa [2]. The region once shared a history of low availability of salt [3]. Also, industrial and commercial complexes that are also centers of economic and job opportunities are concentrated in cities Europeans developed during the colonial period [4]. Recently, hypertension, once a rare problem, is now a major public health concern in West Africa and throughout sub-Saharan Africa (SSA) [5-8]. While estimates on the number of hypertensive individuals are unknown in West Africa, in SSA, an estimated 74.7million individuals are hypertensive [9]. By the year 2025, the number of hypertensive individuals is projected to increase by 68% to 125.5 million individuals [9]. This statistics raises important questions about how best to mitigate barriers to optimal hypertension control and reduction of attendant cardiovascular risk. Moreover, the magnitude of hypertension in some West African countries, including in-depth knowledge of underlying risk factors is not completely understood [6,10,11]. Furthermore, hypertension is increasing at alarmingly high rates in areas where it was once rare due to many factors including urbanization [particularly with migration from rural to urban areas], changes in dietary habits, ageing of the population and social stress [5,6,11-13]. There is also a paucity of research on adequate systems-level approaches designed to mitigate the growing prevalence of hypertension in the region [12,14]. Indeed, optimal hypertension control in West Africa is influenced by a series of complex systems-level factors whose understanding is crucial for the development of effective interventions [15].

In this review, we examine the prevalence of hypertension in West Africa; discuss factors that influence its diagnosis, treatment and control. Furthermore, we; highlight social and structural determinants of hypertension and thus, the importance of systems thinking for interventions aimed at reducing the prevalence of hypertension in West Africa. For the purpose of this review, systems thinking is defined as a ‘paradigm or perspective that considers connections among different components (or systems), plans for the implications of their interactions, and requires an active engagement of those who have a stake in the outcome to govern the course of change [16]’.

## Methods

Relevant published literature on the prevalence of hypertension in West Africa were identified via searching of the PUBMED, African journals online, and PsycINFO database. The review methodology was adapted from a previous review on cardiovascular risk factor burden in SSA [17]. The search was limited to studies published between 2000 and 2013. Search terms used (see Table 1) related first to “hypertension” and “West African country” of interest (i.e. Benin, Ghana, Nigeria etc), secondly, hypertension outcomes such as hypertension prevalence, awareness, treatment, and control. In addition, we specifically searched for papers focused on the social determinants influencing the prevalence hypertension which we defined as factors such as economic or financial costs, access to health systems, and urban-rural divide that operate differentially across the life course to influence the prevalence of hypertension in the region. Both quantitative and qualitative articles were included. The inclusion criteria were: original research paper that reported community-based hypertension prevalence and/or social determinants influencing hypertension. The following papers were excluded from our review: research papers involving clinical trials, dissertations, and conference reports. In all, a total of 294 articles were retrieved. However, only 42 articles met the inclusion criteria. Also, to deal with the heterogeneous nature of available studies, data was analyzed using principles of thematic analysis [18] to identify, compare and contrast recurring themes across included studies.

**Table 1:** West Africa and hypertension outcomes/social determinants key word search

West Africa	West Africa, plus each country in West Africa was also searched by name: Benin, Burkina Faso, Cape Verde, Gambia, Ghana, Guinea, Guinea Bissau, Ivory Coast, Liberia, Mali, Mauritania, Nigeria, Nigeria, Senegal. Sierra Leone, Togo
Hypertension outcomes/social determinant factors	Hypertension/high blood pressure, prevalence, awareness, treatment, medication compliance, financial costs, access to health care, drugs, urbanization.

### Prevalence of hypertension in West Africa

In 1996, Kaufmann and colleagues noted that the *‘determinants of hypertension in West Africa have not been well defined’*[10]; and the authors concluded that *‘the prevalence of hypertension in West Africa was considerably lower than typically observed in industrial countries’*[10]*.* Currently, estimates from available studies suggest that the prevalence has increased over the past decade and is rising rapidly with an urban-rural gradient that places higher hypertension burden on urban settings compared to rural settings. Available data indicates that the prevalence of hypertension is in line with the degree of urbanization in West Africa, with prevalence highest in Guinea (43.6%), [19] Burkina Faso (40.2%), [20] Nigeria (38.2%), [21] and Togo (36.7%), [22]. Although the prevalence of hypertension in urban settings is higher than in rural settings, hypertension is also increasing rapidly in rural areas as well. Indeed, available data indicates an increase in the prevalence of hypertension particularly in rural settings over time. For example, in Ghana, findings from a 1973 survey in 20 rural villages showed a prevalence rate of 4.5%, [23] while recent studies, more than twenty years later, showed prevalence of 25.4% in the rural Ga District [24] and 35% in rural Adankwame community of Ghana [25].

### Hypertension detection, treatment and control

The challenge of hypertension in West Africa is even more complex due to low levels of detection/diagnosis, treatment and control. As shown in Table 2, findings from available studies indicated that overall levels of awareness of one’s hypertension status are consistently low in West Africa with awareness rates less than 30% in all the countries [26-29]. For example, in a survey of 6853 adults in the Republic of Benin, about 77% were unaware of their hypertension status [26]. Although rates of hypertension treatment range from 28% in Ghana [5,6,27] to over 80% in Nigeria, [28] the rates of blood pressure control were abysmally low with less than 10% reported in all countries surveyed except Nigeria where a rate of almost 30% was reported.

**Table 2:** Difference in awareness, treatment, and control of hypertension in select West African countries

	**Awareness (%)**	**Treatment (%)**	**Control (%)**
Benin [26]	6.9	4.8	1.9
Ghana (Kumasi and Ashanti) [5,6,27]	34	28	6.2
Ghana (Ga district) [24]	26	50	16.7
Nigeria (South-South region) [29]	18.5	77.3	29.4
Nigeria (Northern region) [28]	13.9	85.7	12.5

### Social determinants of hypertension

In the WHO seminal work on ‘Equity, Social Determinants and Public Health Programmes,’ Mendis and Banerjee [30] noted that factors such as exposure to lifelong behavioral risk factors such as tobacco use, physical inactivity, unhealthy diet, coupled with job stress, low health seeking behaviors, and less access to medical care operate differentially across the life course to influence the prevalence of cardiovascular risk factors.

### Structural and economic determinants

In West Africa, available quantitative analysis indicate that poverty is rife with more than half of its population (60%) living on less than one dollar a day [31]. While the exact mechanisms through which poverty influences hypertension is unknown, available evidence indicate that structural and economic determinants related to conditions of poverty such as insufficient finances have a direct impact on adherence to prescribed antihypertensive medications [32-34]. The World Health Organization report on medication adherence to chronic diseases suggests that poor medication adherence is a serious problem leading to compromised health benefits and significant economic consequences in terms of wasted time, money and other resources [35]. Mendis and colleagues noted that *‘the fact that most patients have to pay out of their own pocket for consultation and medications either fully or in part, is likely to have a negative impact on long-term management of hypertension’*[36]. In a practice-based survey of 250 hypertensive patients in Nigeria, Ilesanmi and colleagues reported a significant economic burden of hypertension treatment such that 52.8% of participants in their study were spending a tenth or more of their income on health care related expenses [32]. Findings from a survey and focus group discussion with 440 community residents in southwest Nigeria also indicate that economic determinants such as financial hardship significantly influences treatment compliance [34]. Similarly, high costs of drugs have been shown to be associated with poor adherence to antihypertensive treatments in Ghana [33,34,37]. For example, Buabeng and colleagues (2004) found that in a sample of 128 patients, 93% (119 patients) did not comply with their medications citing unaffordable drug prices as the main reason for non-adherence [33]. In Kumasi, Ghana, out-of-pocket expenditures for antihypertensive therapies was reported as a barrier to antihypertensive medication adherence [37]. This was also the case in 225 hypertensive patients attending a tertiary clinic in Lagos Nigeria, where the authors also noted that lack of finances accounted for 23.8% of non-compliance with antihypertensive drug therapy [38].

### The effects of urbanization and hypertension in West Africa

In 2011, the proportion of West Africa’s population classified as urban reached 44.9% [39]. By 2025, it is projected that 52.7% of West Africa’s population will reside in urban areas [39]. Increasing urbanization typically leads to changing lifestyle factors which manifest in rapid epidemiological transitions with increased chronic disease burden [17] in West Africa. For example, in Dakar, Senegal, Duboz and colleagues, [13] reported that length of sojourn in urban environment (Dakar) was a significant predictor for hypertension. The authors reported that people who reside in the city for less than 10 years have reduced risks of developing hypertension. Armstead and colleagues [40] also noted that ‘urbanization is often marked by challenges that require multiple kinds of acculturative coping’. As a result, westernization, financial stress, redefinition of cultural identity, and movement away from traditional coping mechanisms may produce stress among urban residents thereby contributing to hypertension development. This was the case, in Benin, where Sodjinou and colleagues [41] reported that length of time living in urban residence, independent of age and sex, was associated with a higher risk of hypertension. The authors noted that urbanization exacerbates stress exposure as well as social deprivation, and financial constraints thereby contributing to incidence of hypertension [41]. Among residents of Abuja in Nigeria, Adediran and colleagues [42] found that urbanization significantly contribute to increased prevalence of hypertension and other cardiovascular risk factors. Urban dwellers in their study had higher systolic and diastolic blood pressure rates when compared to age-matched rural dwellers with similar genetic background [42] Similarly, in community-based study among the Igbo’s of Eastern Nigeria, Ekezie and colleagues [43] observed that in both men and women, urban participants showed higher incidence of hypertension when compared to rural participants. This was also the case in Ouagadougou, Burkina Faso where Niakara and colleagues [44] found hypertension to be highly prevalent in the urban area.

### Health infrastructure and healthcare workforce

Throughout sub-Saharan Africa, available evidence indicates that inadequate health infrastructure is a barrier to optimal hypertension control [45]. In West Africa, access to healthcare is limited and as a result hypertensive patients often lack adequate healthcare support with hypertension detection, treatment and control. For example, in southwest Nigeria, Yusuff and Balogun [46] noted that there was no ‘institutional system in place to monitor, detect and document adverse drug reactions among patients on anti-hypertensive drug therapy’. One of the most cost effective means of early identification and management of hypertension is regular blood pressure monitoring. However, lack of or inadequate blood pressure measuring device have been identified as barriers to management of optimal hypertension in parts of West Africa. For example, in Oyo State Nigeria, faulty blood pressure monitoring devices have been cited as reasons for not routinely measuring blood pressure in adults attending health care facilities [36].

In addition to poor health infrastructure, in many parts of West Africa, and sub-Saharan Africa, the shortage of healthcare workforce has reached a crisis point and it severely limits the ability to effectively reduce hypertension-related morbidity and mortality [15]. Addressing healthcare workforce shortage is critical if effective management of hypertension is to be achieved in West Africa [15]. Within a broader intervention to address severe shortage of healthcare workforce must be efforts to address physician-related factors, including indifference towards elevated blood pressure among treated patients and insufficient awareness to treatment guidelines [47,48]. For example in southwestern Nigeria, Ono and colleagues [49] reported failure of primary care physicians to intensify antihypertensive medications. Moreover, they reported a tendency to keep patients on monotherapy or “no drug treatment” even in cases of repeated visits to the clinic. Some of these cases were reported to be patients with uncontrolled hypertension and attending physicians declining to prescribe moderately aggressive combination therapy when patients revisited the clinic.

### System thinking: conceptualizing a model of optimal hypertension control in West Africa

Evidence of social and structural determinants of increased rate of hypertension in West Africa highlight the need for systems-level approaches targeted at the reducing the increasing burden of hypertension in the sub-region. Anderson and Johnson [50] define systems as “a group of interacting, interrelated, or interdependent components that form a complex and unified whole”. The WHO report on Systems Thinking [51] suggests that systems are self-organizing, constantly changing, tightly linked, governed by feedback, non-linear (and unpredictable), history dependent, counter-intuitive, and often resistant to change. These essential characteristics help to explain the dire nature of the evidence of the high burden of hypertension in West Africa. Given that optimal hypertension control depends on multiple factors that go beyond simply modifying the behavior of the individual alone, systems-level approaches provide the opportunity to explore the interplay between the full range of human, social, economic, environmental, and health systems factors that influence the rapidly rising prevalence of hypertension in West Africa. Adoption of a systems-level approach also enables the development of an in-depth understanding of the complex factors that influence optimal management and control of hypertension in West Africa. By optimal management and control of hypertension, we mean availability and accessibility of blood pressure monitoring measures to the population. Within the context of a system, for example, optimal hypertension control involves actors such as stakeholders (patients, family members, healthcare providers, community health workers, policymakers etc.), access to services (blood pressure monitoring devices, primary health care clinics, access to antihypertensive drugs) and actions (knowledge and awareness, treatment compliance, linkage to primary healthcare practices, health education etc). Each actor and relationship may have diverse interests, but the common goal would be in increase awareness of hypertension prevalence and preventive measures like blood pressure monitoring thus contributing to the complex system of optimal hypertension control.

### Proposed model: causal loop diagram of optimal hypertension control in West Africa

**Figure 1:**
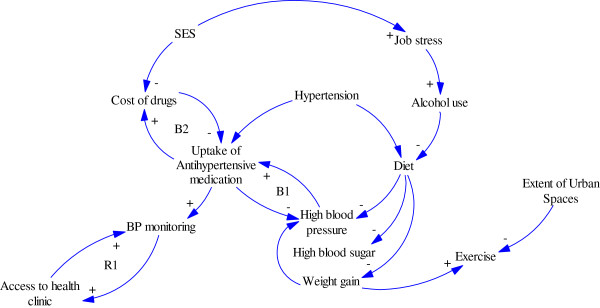
Causal loop diagram of multiple factors influencing optimal hypertension control in West Africa.

Casual loops provide a visual means of articulating our understanding of the relationship/connections between the most salient variables [52,53] that affect a given behavior or problem. They also provide the opportunity to explore the anticipated and unanticipated effects of intervening on these variables as well as the impact of change from one variable to another [52,53]. Drawing from the review of literature on the social determinants and barriers to optimal hypertension control in West Africa, Figure 1 (adapted from Strumberg [54]) depicts an example of a causal loop diagram illustrating the multiple factors influencing optimal hypertension control in West Africa. The text in the figure indicates selected systems-level variables; while the curved arrows depict the relationship between the variables as well as the direction of influence. The addition of positive and negative signs on the arrow describes the relationship between the variables with a positive sign indicating that an increase in one variable causes an increase in the other variable such that change is in the same direction. A negative sign indicates that a change in the variable will cause a change in the other variable, but in the opposite direction. Feedback loop occurs when variables, through a set of other variables, is connected back to itself. A feedback loop may reinforce a situation (R) representing a growth or declining action or balancing it by neutralizing or self-regulating action [52]. As depicted in Figure 1, the proposed causal loop diagram illustrates possible systemic effects on optimal hypertension control with the curved arrows and the +/- symbols illustrating the direction of influence between the variables. Thus, for example, environmental stress such as increased job stress and alcohol use both may lead to poor dietary habits, which in turn may lead to increased BP levels. Similarly, as costs of drugs increases, patients are unable to afford prescribed antihypertensive medication with resultant poor medication adherence, which in turn leads to increased BP levels. Compliance with recommended antihypertensive medications due to elevated BP levels is also influenced by the high costs of the drugs hence the balancing feedback loop labeled B1. At the bottom of the diagram, both access to primary healthcare practices/clinics and the extent of urban spaces are part of a more extensive feedback mechanism that acts in multiple ways to influence increased BP levels. This diagram is complex enough to highlight why systems-level approaches matters when identifying leverage points for the reduction of hypertension-related morbidity and mortality in West Africa. Furthermore, the diagram depicts how changes in one or more variables may have large and potentially non-linear effects on another variable which together influences optimal control of hypertension in the sub-region.

## Conclusions and implications

In this review, we explored the increasing prevalence of hypertension in West Africa; identified the social and structural determinants influencing the prevalence; and highlighted the potential role of systems-thinking approaches to achieving optimal control in the sub-region. In West Africa, there is ample evidence to suggest that the prevalence of hypertension has increased dramatically over the past decade. Hypertension, once rare in rural parts of West Africa, has now emerged as a critical health concern and the trajectory is upward and factors are complex. For example, recent findings from Ghana report a high prevalence of hypertension among rural agrarian farmers that may not be easily attributed to western diet given their reliance on traditional home-cooked meals [55]. It was suggested that differential exposure to stress may trigger a chain of neuroendocrine events cumulating in a high prevalence of hypertension [55]. If this is true, then such differential exposure to stress would add a molecular component to the many other social and structural determinants of hypertension in West Africa. In another example, in Benin, Sodjinou and colleagues [41] noted that long-term residence in urban areas increases the risk of hypertension. Although the authors did not collect data on stress, they suggested that ‘social deprivation, financial constraints and the pressure associated with urban living add to the risk of hypertension [41]’. Furthermore, other studies from the region also suggest that the differential health and health care outcomes are associated with the rising incidence of hypertension in the sub-region.

The adverse impact of stress, coupled with urbanization and weak health infrastructure provide a good rationale why systems-thinking approaches could be essential for the effective management and optimal hypertension in West Africa. This is particularly important in the context of recent advances in hypertension management including new therapeutic options and innovative solutions to expand health workforce so as to meet the high demand for healthcare. Although these opportunities signal optimism about improved hypertension control, the success of these strategies will rely on a new understanding of the complexity of human behaviors and interactions most aptly framed from a systems-thinking perspective. Indeed, a patient’s ability to effectively manage and control their hypertension is a product of a dynamic process, influenced by multiple social, structural, economic and environmental factors that change and interplay over time. Therefore, simply put, promising advances in therapeutic options or task-shifting to cope with the high demand for care could be futile, if the same vigor is not applied to understanding the complex nature of hypertension control and how effective case-management depends on multiple factors that go beyond simply the behavior of the individual alone or the growing shortage of healthcare workforce.

This review has several implications. First, it is important to explore the social determinants of optimal hypertension control in West Africa. As evidenced in the literature reviewed, pragmatic considerations of the root social factors such as poverty, is critical for understanding factors influencing uptake of services and the development of cost-effective approaches to optimal hypertension control. Second, to the extent that future studies and policymaking seek to identify ways to reduce the increasing prevalence of hypertension in the sub-region, knowledge of differential access to healthcare as well as differential exposure to multiple factors ranging from the adverse impact of globalization and urbanization to changes in dietary patterns and constraints with adopting health behaviors are crucial. Third, systems-thinking methods can contribute innovatively to developing an in-depth understanding of the complex factors that influence optimal control of hypertension in West Africa ranging from the role of job stress, to high costs of antihypertensive drugs, coupled with unhealthy dietary habits, and inadequate access to effective blood pressure monitoring devices and primary health practices. The application of systems-thinking modeling like causal loop diagrams illustrates not only the causal factors at play but also leverage points where optimal hypertension control in West Africa can be achieved. More importantly, it enables researchers and policymakers to view the rising prevalence of hypertension in West Africa as a complex health problem composed of different, interacting components and emphasizes the importance of understanding the behaviors of the whole components rather than individual components. Despite these implications, there are some potential limitations with this review as well as challenges or barriers in using systems thinking to findings solutions to optimal hypertension control in West Africa that should also be noted. Differences in study populations, including small numbers of participants in some studies and inconsistencies in the definition of hypertension outcomes limits confidence in conducting a thorough systematic review of available literature. Publication bias is also possible as available studies varied widely in the methodologies used. Partly to deal with the heterogeneous nature of available studies, we used principles of thematic analysis to identify, compare and contrast themes relating to the multiple factors influencing hypertension prevalence in West Africa. Also, with reference to systems thinking approaches, we anticipate that long-entrenched traditions with care-seeking alongside narrow focused and/or regimental attitudes in health service delivery from practitioners may act in various ways to influence optimal control of hypertension in the region. Furthermore, implementation of systems thinking approaches may also be affected by a range of factors that can be barriers or facilitators to optimal management of hypertension. Nevertheless, the rising prevalence of hypertension in the region require solutions that go beyond simply modifying the behavior of the individual patient alone to address the multifactorial nature of the barriers to optimal hypertension control. Simply put, developing interventions to reduce the increasing prevalence may be futile, if attempts are not made to understand, for example, the social, environmental, and health systems contexts in which patients and providers interact for diagnosis, treatment and management of an illness. Taken together, in light of the pressing need to reduce hypertension-related morbidity and mortality rates not only in West Africa, but throughout sub-Saharan Africa, these findings suggest the need to further explore the exact mechanisms through which social determinants influence optimal hypertension control while modeling the potential pathways and entry points where reduction in BP levels can be achieved in West Africa.

## Competing interests

The author declares that they have no competing interests.

## Authors’ contributions

JI had the original idea for this paper which was refined by CA and GB. CA and GB were involved throughout in supervising the writing of the paper and in discussing the findings. RC, BT, JPR, and RA provided comments on various drafts and contributed to writing the final manuscript. All authors have read and approved the final manuscript.
